# The feasibility and impact of embedding pedagogical strategies targeting physical activity within undergraduate teacher education: *Transform-Ed!*

**DOI:** 10.1186/s40814-019-0507-5

**Published:** 2019-11-07

**Authors:** Natalie Lander, Harriet Koorts, Emiliano Mazzoli, Kate Moncrieff, Jo Salmon

**Affiliations:** 1 Faculty of Arts and Education, School of Education, Deakin University, Geelong, VIC Australia; 2 Institute for Physical Activity and Nutrition Research, Deakin University, Geelong, VIC Australia; 3 Faculty of Health, School of Health and Social Development, Deakin University, Geelong, VIC Australia

**Keywords:** Pre-service teacher education, Initial teacher education, Physical activity, Sedentary behaviour

## Abstract

**Purpose:**

Low levels of physical activity and high levels of sedentary behaviour are pervasive, especially in schools. Pre-service teacher education is pivotal to school and educational reform but is an under-studied setting for physical activity and sedentary behaviour intervention research. The objective of this pilot study was to test the feasibility and potential impact of embedding evidence-based active pedagogy based on an adapted version of *Transform-Us!*, *Transform-Ed!* in one core unit of an undergraduate teacher education degree.

**Methods:**

Baseline and follow-up measures (i.e. surveys) were conducted with Bachelor of Education (Primary) pre-service teachers who received the *Transform-Ed!* intervention and academic educators who delivered the intervention. Focus groups of senior academics and telephone interviews with primary school principals examined perceptions of intervention feasibility and explored potential real-world relevance and impact of pre-service teachers training in active pedagogy.

**Results:**

After 12 weeks, pre-service teachers (*n* = 218) were significantly more willing (*pre–post change* Δ = 0.54, 95% CI [0.16, 0.91]), confident (Δ = 1.40, 95% CI [0.89, 1.91]) and competent (Δ = 2.39, 95% CI [1.85, 2.92]) to deliver *Transform-Ed!*, had more positive feelings about the impact of physical activity on student outcomes (Δ = 2.05, 95% CI [1.58, 2.52]), and perceived fewer barriers to integrating *Transform-Ed!* into current and future teaching (Δ = − 7.26, 95% CI [− 8.88, − 5.64]). Four major themes emerged from the focus groups (*n* = 9) and interviews (*n* = 5) around participant perceptions of *Transform-Ed!*: (i) acceptability and appropriateness, (ii) need (tertiary level), (iii) need (primary level) and (iv) overcoming challenges.

**Conclusion:**

The *Transform-Ed!* pilot study demonstrated promising results across multiple participant levels, as it was perceived to be feasible, acceptable and appropriate by pre-service teachers, academics and school principals. The findings have direct implications for the progression of *Transform-Ed!* from pilot to a future definitive trial.

## Background

Physical activity plays an important role in the prevention of metabolic, cardiovascular, musculoskeletal and mental health risk factors in children [1]. In addition, physical activity has been positively associated with increased ‘academic performance’ [2–4], including cognitive skills (e.g. executive functioning, attention, memory, comprehension) [5], attitude (e.g. motivation, self-concept, satisfaction) [6], academic behaviour (e.g. on-task time, organisation) [4] and academic achievement (e.g. standardised test scores, classroom tests) [7]. Despite this, only 14% of Australian children (5–12 years) meet government-recommended levels of 60 min of physical activity per day [8, 9]. Improving opportunities for physical activity engagement across the school day remains a global research priority.

Emerging evidence suggests that, independent of physical activity, prolonged sitting adversely affects children’s health [10–12]. Sedentary behaviour has been associated with lower fitness, unfavourable body composition, cardio-metabolic risk, lower self-esteem and poorer health later in life [13, 14]. To reduce these health risks, recent government recommendations suggest that youth should minimise sedentary time and break up sitting time as often as possible [8, 9]. Despite these recommendations, school children spend around 70% of their day sitting [15].

Schools can play *a part* in the physical activity behaviours of youth, as they have access to most children regardless of race, ethnicity or socioeconomic status and for many hours on weekdays [16]. Traditionally, physical education classes, school sport and recess have provided opportunities for physical activity. Yet, reduced offerings at recess and reductions in physical education scheduling suggest that the school environment is becoming increasingly inactive [16]. Approaches to maximise children’s daily physical activity are therefore vital, such as classroom-based physical activity (i.e. active teaching) [7], including active lessons (e.g. learning maths by stepping or jumping), active breaks (e.g. ‘stand and discuss three key things you have just learned’) or physical activity curriculum content (e.g. lessons on physical activity skills or knowledge).

*Transform-Us!* [17] was a successful 18-month, four-arm cluster-randomised controlled trial in 20 primary (elementary) schools, with over 220 teachers and 1600 students, in Melbourne, Australia. The study aimed to increase children’s physical activity and decrease sedentary behaviour across the school day by incorporating a mixture of educational, pedagogical, behavioural and environmental approaches in order to integrate movement into everyday class lessons, recess/lunchtime and homework [17]. Results demonstrated numerous positive student outcomes such as reduced sitting, increased moderate-to-vigorous physical activity, and lower body mass index, waist circumference and systolic blood pressure [18]*.* In contrast, other school-based physical activity interventions have had variable results [11], only reporting small effects (e.g. 4 min more activity per day) [19]. Potential reasons for these disappointing outcomes include poor delivery, poor uptake or variable program adherence [20]. Although *Transform-Us!* was highly successful, it may not be possible to continue delivering face-to-face professional development to in-service teachers, as it is time, resource and cost intensive and thus perhaps not sustainable. Integrating active teaching pedagogy into initial or pre-service teacher education may provide a more effective and potentially more sustainable approach.

Pre-service teacher education programmes aim to prepare graduates to become quality teachers equipped with pedagogical practices that will serve to meet the increasing demands associated with the teaching profession [21]. Indeed, pre-service teacher education provides an integral platform for scaffolding critical pedagogical skills, strategies, knowledge and capabilities [22], and as such is viewed as a crucial link in producing quality in-service teachers and more positive student outcomes [21]. The impact of pre-service teacher education on teachers’ effectiveness and students’ outcomes is internationally recognised as pivotal [22], yet it is an under-studied and perhaps under-utilised setting for physical activity and sedentary behaviour intervention research.

Therefore, the objective of this pilot study was to test the feasibility and potential impact of embedding evidence-based active pedagogy (*Transform-Ed!)*, based on an adapted version of the efficacious *Transform-Us!* program, into one core unit of an undergraduate teacher education degree. Subsequently, the study aimed to evaluate the merit in progressing the *Transform-Ed!* intervention from pilot to future definitive trial. The specific aims were to (i) assess pre-service teachers’ perceptions of implementing *Transform-Ed!* strategies into current and future teaching practice; (ii) investigate changes in academic educators perceived confidence, self-efficacy and implementation in delivering the activity pedagogy; and (iii) investigate perceptions of feasibility and potential impact of *Transform-Ed!* among senior academics and principals.

## Methods

### Design

A mixed method pre-post pilot study was conducted to examine the feasibility and potential impact of embedding *Transform-Ed!* within one core unit of an undergraduate teacher education degree. Where relevant, the CONSORT checklist for pilot trials informed the study design [23]. Baseline and follow-up measures (i.e. surveys) were conducted with Bachelor of Education (Primary) pre-service teachers (i.e. generalist elementary pre-service teachers of children between the ages of 5 and 12 years) who received the 12-week *Transform-Ed!* intervention and academic educators (i.e. lecturers or tutors) who delivered the intervention. Focus groups (FGs) were conducted with a sample of senior academics in the School of Education, and telephone interviews were conducted with a sample of primary school principals to examine their perceptions of intervention feasibility and to explore potential real-world relevance and impact of pre-service teachers trained in active pedagogy. The study was approved by Deakin University Human Ethics Committee (HAE-17-207).

### Recruitment and consent

To maximise participant uptake, all Bachelor of Education (Primary) pre-service teachers enrolled in the core unit ‘Introduction to Curriculum and Pedagogy’ were invited to participate in the study (March 2018) via the University unit cloud site (online platform); direct emails were sent to students’ university email accounts; and hard copies of the study advertisement via flyers were distributed around the university campus. A plain language statement was provided to all students, and signed consent to participate was required prior to the first *Transform-Ed!* session and the completion of surveys. To assess survey test–retest reliability, participants were invited to complete the same survey 1 week later.

Academic educators (i.e. tutors and/or lecturers responsible for the delivery of the curriculum and pedagogy unit, the target unit for the *Transform-Ed!* program) were invited to participate in *Transform-Ed!* (i.e. professional development, intervention delivery and pre/post surveys) via email invitation. Senior academics were invited to participate in FGs via email invitation. The senior academics are crucial decision makers and gatekeepers in regard to course and unit design, structure, curriculum, modes of delivery, assessment and policy. As such, an understanding of the views of these key stakeholders not only helps to inform the feasibility of the intervention, but is also integral to the development of a future definitive trial. The senior academics invited into the study included: Director of Research (School of Education), Head of Teaching and Learning (School of Education), Course Director (Bachelor of Education: Primary), Unit Chair (Curriculum and Pedagogy), Course Direction (Health and Physical Education), Unit Chair (Mathematics and Children) and Unit Chair (Literacy Teacher Learner).

**Fig. 1:**
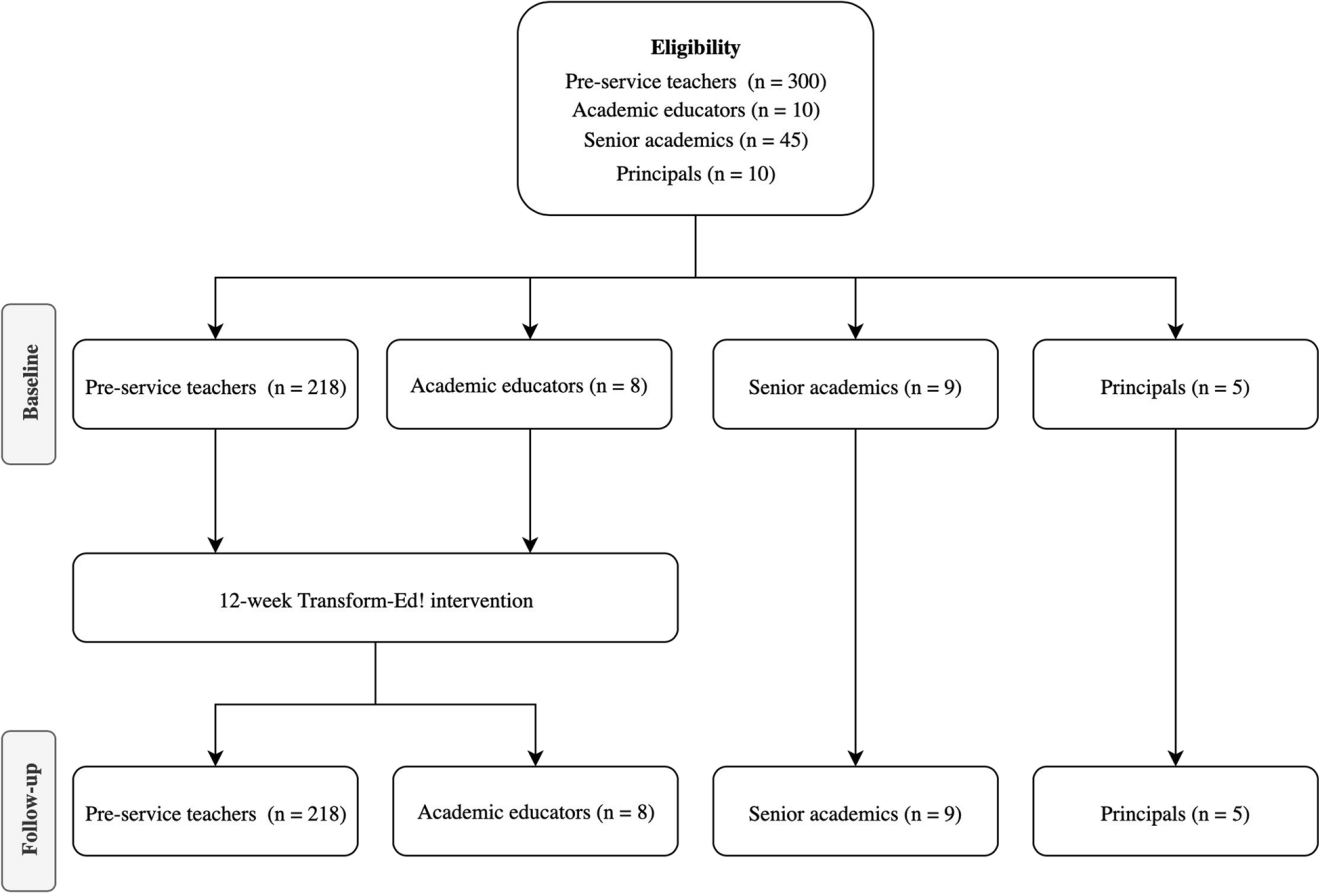
Participant flow

Principals of primary (elementary) schools within a 15-km radius of the university were invited, via email invitation, to participate in telephone interviews. These primary schools are frequently used as placement schools for the pre-service teachers and are potential employers of university graduates. The principals form a relevant and important group of stakeholders, who can provide researchers with real-world information they need to develop programs to facilitate implementation and subsequent compliance. The school-based stakeholders (i.e. Principals) were interviewed to identify program feasibility as well as the potential real-world relevance and impact of hosting placement teachers or employing graduate teachers trained in active pedagogy. Phone calls were made to follow-up the invitations to academic educators, senior academics and principals, and interested participants were provided with a plain language statement and a written consent form (see Fig. 1 participant flow).

### Intervention

*Transform-Ed!* was embedded into one core curriculum and pedagogy unit of the Bachelor of Education (Primary) degree. The targeted unit is the first curriculum and pedagogy units in a series of eight scaffolded units across the 4-year degree. It introduces core elements of the primary (elementary) curriculum and relevant pedagogies, drawing on examples from health and physical education, literacy and mathematics. It is a 12-week unit, including 12 1-h lectures and 12 2-h practical-based seminars. *Transform-Ed!* strategies were developed by the lead researcher, who is an experienced lecturer in Education, in conjunction with *Transform-Us!* chief investigators, the course director of Bachelor of Education (Primary) and the existing unit chair of the selected curriculum and pedagogy unit. The *Transform-Ed!* intervention content was guided by the original *Transform-Us!* content, which was framed by social cognitive theory [24], behavioural choice theory [25] and ecological systems theory [26]. An overview of the theoretical basis of the adapted version of *Transform-Us!* (i.e. *Transform-Ed!)* and links to program objectives are presented in Additional file 1. In brief, these theories (i.e. social cognitive theory [24], behavioural choice theory [25] and ecological systems theory [26]) have previously been shown to be effective in encouraging behaviour change in children’s physical activity and sedentary behaviour. They recognise that there are multiple levels of influence on health behaviour including intrapersonal (e.g. awareness, self-efficacy, enjoyment), interpersonal (e.g. parents & teachers), physical environmental (e.g. classrooms and playgrounds) and policy influences (e.g. school and classroom policies) [17]. In light of this, the *Transform-Ed!* intervention content included learnings around three key areas, namely (i) classroom-based physical activity/active teaching (e.g. physically active academic lessons, active breaks, health-based curriculum content), (ii) active environments (e.g. encouraging activity at recess and lunchtime) and (iii) active families (e.g. engaging families via active homework). The key messages were disseminated to pre-service teachers, by the academic educators, in the following ways: modelling active teaching and active breaks throughout lectures and seminars; providing the theoretical underpinnings of the importance of physical activity; delivering education around pedagogies that are designed to promote activity; facilitating peer micro teaching opportunities, whereby the pre-service teachers practice active teaching strategies and receive peer and lecturer feedback; and comprehensive lesson plan resources on active teaching, active breaks and active homework. Examples of the *Transform-Ed!* content and how this was embedded by academic educators is provided below in Table 1.

**Table 1:** Examples of how *Transform-Ed!* key messages and active teaching strategies were embedded into the ‘Introduction to Curriculum and Pedagogy’ unit of the Bachelor of Education (Primary) degree

Strategy	Elaboration	Strategy	Examples
Active academic lessons	• Normal planned lessons, where the delivery method rather than the content is changed.	• The lecturer modelled how to increase physical activity and decrease sedentary behaviour in primary class lessons by integrating active teaching strategies into lectures and practical seminars. • The pre-service teachers were educated (pedagogical theory) around the skills, strategies, organisational and managerial concepts required to teach actively. • Pre-service teachers were provided with a comprehensive resource of active teaching examples/exemplars. • Pre-service teachers were provided the opportunity to practice skills, strategies, organisational and managerial concepts required to teach actively, and received peer and lecturer feedback on their performance.	• Active numeracy: Measure that space: students physically measure the size of a large area and/or objects in that area with a range of tools and describe the area in terms of formal (e.g. tape measure, ruler etc.) and informal measurements (e.g. piece of A4 paper, string, cricket bat, steps etc.). Students begin by making or finding ‘measuring tools’, then measure the space and/or object, using both sets of devices and record, tabulate then discuss the measurements. • Active literacy: All students stand. The teacher gives all of the students a ‘part of speech’ (e.g. noun, pronoun, verb, etc.) to focus on. The teacher reads sentences or a passage aloud, and each time that part of speech is used, the students perform an exercise that has been chosen by the teacher or students (e.g. star jump, hop, squat etc.)
Active breaks from sitting	• During extended teaching blocks, short active breaks were used interrupt prolonged periods of sitting.	• The lecturer modelled how to increase physical activity and decrease sedentary behaviour in class lessons, by integrating active beaks into lectures and seminars. • The pre-service teachers were educated (pedagogical theory) around the skills, strategies, organisational and managerial concepts required to incorporate active breaks into teaching. • Pre-service teachers were provided with comprehensive resources related to active breaks. • Pre-service teachers were provided the opportunity to practice skills, strategies, organisational and managerial concepts required to break sitting time and received peer and lecturer feedback on their performance.	• Stand and discuss (stand up and tell your partner about one new thing you just learned) • Stand up and ‘move like a …’ for 30 s (categories may include the natural environment (e.g. oceans, earthquakes); modes of transport, animals, etc. • True or false: Allocate one side of the classroom as ‘true’ and the opposite as ‘false’. Ask students a question, the students who believe the answer is true move to the ‘true’ side and the students who believe the answer is false move to the ‘false’ side.
*Transform-Ed!* health lesson curriculum content	• Class lessons, which aim to build skills and increase knowledge about the importance of being active and sitting less.	• Pre-service teachers were are educated around the importance of adequate physical activity, with a particular focus on physical activity and school children/school environment. • Pre-service teachers provided with comprehensive resources for future teaching around the importance of physical activity. • Pre-service teachers provided opportunity for micro teaching (peer teaching) in regard to delivering activities and lessons around physical activity. Pre-service teachers received peer and lecturer feedback around their performance.	• Stencil yourself: Students work in groups and stencil around one group member on a large piece of butcher’s paper. The students brainstorm how physical activity affects them and record the ideas on the stencilled paper. Students are encouraged to include physical, social, cognitive and psychological influences. Students then move around groups, learning from others and adding suggestions where appropriate.
Active environments/promoting activity during recess and lunchtime	• Signage/posters, equipment/facilities and teacher encouragement promoting physical activity at recess and lunchtime.	• The lecturer delivers a seminar on playground-based activities that could be used by pre-service teachers to increase primary students PA at recess/lunchtime. • Pre-service teachers provided with comprehensive resources around playground based activities. • Pre-service teachers provided opportunity for micro teaching (peer teaching) in regard to delivering playground based physical activity. Pre-service teachers received peer and lecturer feedback around their performance.	• Using existing asphalt playground line markings around school to encourage movement. • Providing tubs of sports and novel PA equipment (e.g. ribbons, hula-hoops, balls, juggling batons etc.) in each classroom for student use at lunchtime and recess. • Teacher ‘on duty’ encouraging and modelling active play.
Engaging families	• Newsletters and activities provided for parents and children to engage with to reinforce the importance of children being active and sitting less.	• The lecturer delivered a seminar on active homework strategies that could be used by pre-service teachers to engage families around the importance of increasing PA and decreasing sitting time at home. In addition, pre-service teachers were educated around the importance of engaging families and the community when addressing physical activity behaviour (e.g. ecological model). • Pre-service teachers provided with comprehensive resources around active homework examples/exemplars. • Pre-service teachers provided opportunity for micro teaching (peer teaching) in regard to developing and delivering active homework.	Setting children active homework encouraging collaboration with all family members, for example: • Calculate the number of right angles as you walk through from your front door to the backdoor. • Using stride length as a reference, which is the largest room in your house? • Think of all your ‘free time’ at home. How do you spend this time? Are you physically active (in other words, do you move your body) or do you sit down a lot? Create a table and add up your active time and sitting time. As a family think of strategies you could put in place to decrease the amount of sitting and increase the amount of physically active ‘free time’

The pedagogy for *Transform-Ed!* was framed by transformative education [27]. Transformative education suggests that learning is understood as a process of using a prior interpretation to construe a new or revised interpretation of the meaning of one’s experience to guide future action [28]. Specifically, transformative education is teaching and learning that effects a change in perspective [29], which might be an especially relevant approach in relation to initial teacher education around increasing physical activity and decreasing sedentary behaviour across the school day. The baseline survey (as explained below in procedures) collected data on the pre-service teachers’ own experiences and observations of active teaching/learning when they were students. In line with the transformative values of reframing education [29], this data was used not only as baseline data by the researcher, but also as diagnostic assessment by the academic educators, to identify the explicit learning experiences and capability deprivations of the pre-service teachers. Specifically, the pre-service teachers who had recorded the most negative active learning experiences as a student were provided with the most comprehensive education, opportunity and experience around active teaching [29].

Training of academic educators delivering the *Transform-Ed!* intervention was framed by the key characteristics of teacher training for effective physical activity interventions [30] and occurred prior to commencement of the unit (February–March 2018). Training included the following: (i) a 2-h face-to-face workshop, (ii) provision of comprehensive session lesson plans including active teaching content and pedagogical material and (iii) ongoing email/phone/face-to-face support. The *Transform-Ed!* strategies were then embedded, by the academic educators across the 12 1-h lectures and 12 2-h seminars. The curricular content of the unit remained unchanged (i.e. unit learning objectives and assessments). The focus was on the environmental and behavioural influences on physical activity, specifically targeting the academic educators’ delivery methodology and pedagogy of the unit content. Table 1 describes examples of the *Transform-Ed!* approaches that were embedded into the teaching and learning of the unit.

### Procedures

Pre-service teachers completed baseline (March 2018) and follow-up (June 2018) surveys to measure changes in their perceived willingness, confidence, competence and challenges to integrating *Transform-Ed!* strategies into their current and future teaching practice, pre- and post-intervention (12 weeks). The survey was a modified version of the Morgan and Hansen survey [31], which was originally used to assess pre-service teachers’ competency to deliver Physical Education classes, and had been pilot tested with primary school pre-service teachers [31]. The *Transform-Ed!* survey assessed pre-service teachers’ (i) willingness to integrate active teaching into professional practice (teaching) placements, (ii) perceived impact of increased activity and breaking up sitting time on student outcomes, (iii) confidence to integrate specific active teaching strategies within the classroom, (iv) confidence to integrate specific active teaching strategies beyond the classroom (i.e. during recess), (v) competence to effectively integrate specific active teaching strategies across the school day and (vi) perceived barriers to the delivery of active lessons. Questions ranged from five to 15 questions per construct. For example, for ‘perceived confidence to integrate specific active teaching strategies within the classroom’,, pre-service teachers rated their level of perceived confidence to the following: (i) integrate movement into class lessons, (ii) deliver active breaks, (iii) deliver curriculum content on the importance of increasing physical activity and sitting less, (iv) promote active transitions and (v) model active practices within the classroom. Survey responses were based on a 5-point Likert scale (1 = strongly disagree to 5 = strongly agree). Questions within each construct were summed to create total construct scores (range 25–75). Data collected from a sub-sample of pre-service teachers at baseline and 1 week after was used to assess the instrument test–retest reliability. In addition, the baseline survey also asked pre-service teachers to rank their own experiences and observations of active teaching/learning when they were a student, as literature suggests this is a highly influential factor on teacher identity and teaching quality [31].

The academic educators completed brief baseline and follow-up surveys to investigate changes in their perceived confidence, self-efficacy and implementation in delivering the activity pedagogy. The survey consisted of six questions (as shown in Table 3), and each question was scored using a 3-point Likert scale: 1 = disagree, 2 = neither agree nor disagree, 3 = agree.

Focus groups and telephone interviews with senior academics and school principals, respectively, explored their perceptions of the appropriateness, acceptability, potential impact and barriers/facilitators of widespread integration, sustainability and institutionalisation of *Transform-Ed!* (FG/telephone interview guide is presented in Additional file 2). All FGs and telephone interviews were conducted by the lead author. Interview questions and prompts were developed to guide the interview, clarify ambiguous statements and encourage the interviewee to expand on their answers [32, 33]. Focus groups and interviews ranged in duration from 18 to 45 min and were audio-recorded and transcribed verbatim by the lead author to ensure consistency [34]. ‘Member checking’ was performed during the interviews by summarising and relaying participant information to establish accuracy [33], and each participant was emailed their transcript and invited to comment and confirm accuracy (no participant requested changes to transcripts).

### Data management and statistical analysis

Descriptive statistics were used to summarise pre-service teacher sample demographics. Sum scores were calculated for each construct within the survey. Paired *t* tests were conducted to compare pre-service teachers’ willingness, feelings, confidence, competence and perceived barriers total scores before and after the *Transform-Ed!* intervention. All Bachelor of Education (Primary) pre-service teachers enrolled in the core unit ‘Introduction to Curriculum and Pedagogy’ were invited to participate in the study (*n* = 300). Assuming small–medium effects to be found, a conservative estimation conducted using G*Power [35], parameters set at *d* = 0.25, two-tailed and α level = 0.05, suggested that a sample of 210 participants was necessary in order to detect statistically significant differences with paired *t* tests. To assess test–retest reliability, absolute agreement intraclass correlation coefficients, using two-way mixed models, were calculated for the total score of each construct. Due to the small participant numbers of academic educators delivering *Transform-Ed!* (*n* = 8), changes in perceived competence, confidence and willingness to integrate *Transform-Ed!* into the curriculum, as assessed via surveys, was reported descriptively. An inductive content analysis [33] was manually performed to examine the perceptions of *Transform-Ed!* among senior academics and primary school principals. Specifically, a systematic thematic data analysis process to generate categories and explanations, and thus produce the best qualitative evidence, was conducted [32, 33]. Firstly, interview transcripts were reviewed multiple times to facilitate data immersion. Open coding was conducted on all interview transcripts. Descriptive labels were written in the transcript margins, prompting systematic judgments about each segment of text within the data set. As the topics evolved, new codes were added and existing codes were revised and refined to ensure depth and validity of the analysis process. The labels that shared like/similar values or relationships were sorted into clusters, creating categories or themes, which facilitated interpretation of response patterns [32].

## Results

### Pre-service teachers

In total, 218 pre-service teachers (76% females) completed both pre- and post-test measures. The pre-service teachers’ age ranged from 17 to 47 years, with the majority (71%) aged between 17 and 21 years old. Most were in their first year of a teacher education degree (64%), with fewer in second year (35%) and only two participants in their third or fourth year.

The pre-service teachers’ personal experience of active teaching, active breaks and active homework during their own schooling years was collected via the baseline survey and is presented in Additional file 3. In brief, the pre-service teachers rarely experienced active teaching/learning as a student. No participant recalled a classroom teacher incorporating activity into their classroom teaching, nor providing active homework opportunities. The majority did not recall movement being incorporated across the school day outside of physical education class, breaks in prolonged sitting time or the importance of physical activity being conveyed to them in lessons other than physical education. Less than one fifth of the participants recalled activity being promoted at recess and lunchtime.

The mean changes in pre-service teachers’ perceived willingness, competence, confidence and barriers to implementing *Transform-Ed!* strategies are presented in Table 2. There was a significant increase in the total scores related to pre-service teachers’ willingness to integrate active teaching and positive perceptions of activity on student outcomes, as well as their active teaching confidence and competence. There was also a significant reduction in the perceived barriers to the implementation of active teaching strategies. Table 2 also presents the test–retest reliability of the survey instrument. The results showed that the questionnaire was highly reliable for total scores of all constructs [ICC range = 0.89–1.00; 95% CI range 0.85–1.00].

**Table 2:** Changes in first year pre-service teachers’ perceived competence, confidence, willingness and barriers to implementing Transform-Ed! strategies into teaching practice

	Test–retest reliability (*N* = 119)		Mean (SD)		Paired differences: follow-up–baseline	
Construct	ICC	[95% CI]	Baseline (*N* = 218)	Follow-up (*N* = 218)	Mean	[95% CI]
Willingness to integrate active teaching into professional practice placement	0.89**	[0.85, 0.93]	24.67 (2.91)	25.21 (2.27)	0.54*	[0.16, 0.91]
Feelings about the impact of increasing activity and breaking sitting time on student outcomes (i.e. on task time, interest, academic outcomes)	0.98**	[0.97, 0.99]	24.15 (3.34)	26.20 (2.41)	2.05**	[1.58, 2.52]
Confidence to integrate specific strategies *within the classroom*, to increase PA and decrease sitting time across the school day—as future teachers	1.00**	[1.00, 1.00]	20.10 (3.56)	21.50 (2.54)	1.40**	[0.89, 1.91]
Confidence in specific strategies—*beyond the classroom* (e.g. recess, lunchtime and home)—to increase PA and decrease sitting time—as future teachers	1.00**	[1.00, 1.00]	17.71 (4.23)	19.25 (3.03)	1.54**	[0.90, 2.18]
Perceived competence to effectively integrate specific active teaching strategies across the school day—as future teachers	1.00**	[1.00, 1.00]	17.73 (3.76)	20.11 (2.28)	2.39**	[1.85, 2.92]
Perceived barriers to the delivery of active lessons in your class	1.00**	[1.00, 1.00]	47.74 (11.23)	40.48 (9.52)	−7.26**	[−8.88, −5.64]

*ICC* intraclass correlation coefficient, *CI* confidence interval, *SD* standard deviation

*p < 0.01; **p < 0.001

### Academic educators

Changes in perceived confidence, self-efficacy and implementation (i.e. adaptation, fidelity, dose) of staff delivering *Transform-Ed!* units (*n* = 8) are presented in Table 3. In brief, following the *Transform-Ed!* program the majority of the staff felt more confident in their ability to integrate active teaching into seminars and lectures. All staff stated that they would regularly integrate movement into future lessons and perceived that breaking sitting time was important. In addition, at follow-up, the staff perceived active teaching as less of a challenge, distraction and disruption to their teaching, student learning and curriculum outcomes than they did at baseline.

**Table 3:** Transform-Ed! intervention deliverers (academic educators): survey

Variable	Baseline			Follow-up		
	Agree	Neither	Disagree	Agree	Neither	Disagree
I regularly incorporate movement within my lectures/seminars	50% (*n* = 4)		50% (*n* = 4)	100% (*n* = 8)		
Increasing students activity and breaking sitting time is important	87.5% (*n* = 7)	12.5% (*n* = 1)		100% (*n* = 8)		
I am confident in my ability to deliver active teaching strategies	25% (*n* = 2)	25% (*n* = 2)	50% (*n* = 4)	75% (*n* = 6)	25% (*n* = 2)	
Active pedagogy is challenging	50% (*n* = 4)	25% (*n* = 2)	25% (*n* = 2)	25% (*n* = 2)	25% (*n* = 2)	50% (*n* = 4)
Active pedagogy detracts from achieving curriculum outcomes	50% (*n* = 4)	25% (*n* = 2)	25% (*n* = 2)	12.5% (*n* = 1)		87.5% (*n* = 7)
Active pedagogy is disruptive to student attention	50% (*n* = 4)	25% (*n* = 2)	25% (*n* = 2)	12.5% (*n* = 1)		87.5% (*n* = 7)

### Senior academics and principals

Four major themes and several subthemes were identified in the FGs (*n* = 9 senior academics) and interviews (*n* = 5 principals). In brief, although some challenges were discussed (e.g. creating change in regard to long-standing lecturing styles), embedding active teaching strategies across the Bachelor of Education (Primary) was unanimously supported by senior academics and school principals. This was primarily due to the perceived reach of the program, firstly at a tertiary level, but ultimately at the primary school level, which would provide access and enable all students to be active and engaged in their learning.

#### Theme 1: Acceptability and appropriateness

##### Subtheme 1: Cross-curricular acceptability and appropriateness

The senior academics and principals agreed that *Transform-Ed!* may provide a unique and much-needed opportunity to link otherwise isolated curriculum areas, units and domains via a common and united pedagogy. These beliefs are echoed in the following statements:

Ultimately, by using the one ‘active’ pedagogy it enables transferability to teaching/learning objectives and shared outcomes. It demonstrates, in practice, true cross-discipline teaching to the pre-service teachers. This is a unique and much-needed shift in teacher education. (Academic 3)

The content appears to be integrated comprehensively into the curriculum framework and did not appear to impede Unit learning objectives nor assessment requirements. This is providing a new teaching tool in the experienced educators’ tool kit as well as demonstrating a cross curricular approach to teacher education. (Academic 2)

##### Subtheme 2: Classroom-based physical activity appropriateness/acceptability

The participants identified that including activity within the classroom expands the reach of physical activity beyond recess, physical education and sport. It was agreed that teaching actively should become part of every pre-service teacher’s ‘tool kit’ and each classroom teacher’s role. The participants highlighted that this would subsequently promote physical activity to all children, not just the sporty kids. The following statements highlight these perceptions:

This program would extend the access of activity to all children – regardless of their sporting ability and their opportunity outside the school. (Principal 2)

… demonstrates that encouraging activity is an important part of their role as pre-service teachers and ultimately as classroom teachers. (Academic 6)

#### Theme 2: Need—tertiary setting

It was unanimously agreed that a program such as *Transform-Ed!* is not only desirable and feasible, but also has the potential to be a sustainable and widespread approach within pre-service teacher education and tertiary education more broadly. This was primarily due to the perceived need for a different and more progressive approach to pre-service teacher education and indeed tertiary education more generally.

##### Subtheme 1: Novel teaching approach

Participants agreed that academic educators should consistently and systematically review the units they deliver to ensure relevance, value and meaning. Integrating a program such as *Transform-Ed!* may encourage academic educators to reflect and evaluate their own practice.

Re-energise lecturers, it is like new learnings for old lecturers. Get them to reconsider the effectiveness of their teaching style. Evaluate how engaging their teaching is. (Academic 1)

Furthermore, academics acknowledged that upskilling academic educators was imperative to ensure the teachers of the future were receiving best practice and best modelling.

Modelling ‘good teaching practice’ is imperative – it demonstrates what and how our new teachers should be learning and then teaching. (Academic 1)

##### Subtheme 2: Teacher’s skill set

A common theme to arise was that the pedagogy and teaching skills included in the *Transform-Ed!* intervention were viewed as a necessary skill set in a highly progressive and constantly evolving workplace.

Embedding these types of teaching skills in the first year of teacher education is a huge ‘value-add’ in regards to teaching capabilities. (Principal 2)

Many of the participants suggested that an approach like *Transform-Ed!* has the potential to enable teachers to connect curriculum, content and pedagogy via a common approach. Participants noted this as an essential skill set to have, to enable teaching and learning to be more authentic and meaningful. In addition, one of the principals described creating potentially more effective and sustainable change via the pre-service teachers disseminating their new learnings in placement settings.

The strategies promoted in Transform-Ed! seem to provide transferrable skills for all classes/domains. The pre-service teachers are also learning to manage and promote an engaging and dynamic learning environment. This is essential as a new (and old) teacher. Your pre-service teachers could come to our schools and lead the change here. This would be creating change from the bottom up. (Principal 5)

#### Theme 3: Need—primary schools

A common theme to emerge was the participants’ descriptions of the reach and potential benefits a program such as *Transform-Ed!* would have on the primary school system and children.

##### Subtheme 1: Reach and access for all

A predominant response was the extensive reach the program would have for all children to be active, not just the sporty ones. The support for institutionalisation of the program was also apparent. In addition, both academics and principals were aware of the need for many school children to move and be active to learn.

This captures the attention and enjoyment of children who ‘need to move to learn’. It allows (and promotes) focused activity breaks for children rather than reprimanding them for moving. (Principal 2)

I would love this to become part of whole school culture. Where it is the ‘norm’ for all students to be active during class time, not to be seated and still. What I would really love is that this approach is part of every school. Perhaps this is the way we can target more, ideally all, children. (Academic 7)

##### Subtheme 2: Benefits of physical activity

Many of the participants agreed that being active resulted in both immediate and sustained benefits.

Both teachers and students would benefit from an active program such as this, probably more than they are aware. (Academic 1)

Several of the principals highlighted the observed improvements in student behaviour and concentration following periods of activity.

Students love moving – if this is focused movement it becomes such a positive motivator. Students seem so much more settled and focused after recess or after PE. Incorporating activity into everyday class lessons can only be beneficial. (Principal 2)

They also commented on the ‘happy and engaged’ culture that is created when classroom teachers encourage active learning.

When students are actively engaged in movement and learning it sets up a positive learning environment. They seem happy and engaged in their learning. (Principal 2)

Many of the principals were aware of the decline in physical activity levels. Most also viewed it as the school’s responsibility to provide physical activity opportunities as many children would otherwise not be adequately active.

There are the sporty kids who have all the support in the world to be active. Then there are the non-sporty kids – the kids who are not active at recess and not involved in after-school sport. These are the ones programs like this would really benefit. (Principal 2)

#### Theme 4: Overcoming perceived challenges and barriers

Although all participants were very supportive of *Transform-Ed!*, several perceived challenges or barriers to the widespread integration of the approach were raised.

##### Subtheme 1: System change

The academics unanimously agreed that creating change at both a tertiary and primary school level, in regard to pedagogy, would be one of the greatest barriers.

Getting lecturers who have been delivering the same content in the same way for several years to change their ways is a huge challenge … good luck. (Academic 4)

Getting lecturers who are not confident movers to be active will be … interesting and challenging. (Academic 4)

In addition, academics raised the notion of potential facilitators such as incentives and enlisting champions, to assist with change.

Training incentives, whether it be time release or financial, I think there will need to be some sort of incentives for lecturers to firstly attend the professional development, and then adhere to the program would be a challenge. (Academic 2)

Perhaps creating Transform-Ed! champions might be a way forward – getting key people on board may be a challenge – but I think it is imperative to widespread integration. (Academic 2)

##### Subtheme 2: Teachers’ workload and time

One of the principals raised concerns around the additional load it may cause to their already overloaded teachers.

Very hard to ask the teachers to do yet another thing in the long list of teacher duties. (Principal 4)

Another principal raised concerns around the authenticity and purpose of the tasks, ensuring the pedagogy was meaningful rather than a time waster.

Need to ensure tasks are meaningful and not time wasters – how do we mandate this. (Principal 1*)*

Despite the challenges raised there was a strong sense of perceived value attached to the program, and participants agreed that once the value of this program was conveyed and experienced, the advantages will rapidly override the barriers.

Change is hard, and there is so often resistance to change, but I think the evidence behind, and the results from this type of program will drive the much-needed change in regards to initial teacher education … (Academic 2)

## Discussion

This pilot study aimed to test the feasibility and potential impact of embedding evidence-based active pedagogy (i.e. *Transform-Ed!*) within one core unit of an undergraduate teacher education degree. The *Transform-Ed!* intervention provided pre-service teachers with a range of educational, pedagogical, behavioural and environmental approaches to target increases in children’s physical activity, such as active teaching, active breaks and active homework strategies. Results showed that *Transform-Ed!* was strongly supported by Bachelor of Education (Primary) pre-service teachers and academics, as well as a sample of primary school principals. The applicability of the pilot trial methods and findings are therefore conducive to a future more definitive trial [23].

The pre-service teachers in this study recalled limited experiences of active teaching, active learning and physical activity provisions across the school day during their own schooling years. This is congruent with previous research investigating pre-service teachers’ own experience in physical education and physical activity and the negative impact the experience has on future teaching [31, 36]. Teachers’ prevailing negative experiences of activity and physical education are of particular concern given that the personal school experiences of classroom teachers are significant predictors of their confidence to teach activity-based curricular and physical education [31] and significantly influence the quality of their teaching in these areas [36]. The pre-service teachers bring with them a set of beliefs that constitutes their emerging sense of teacher identity, and this is directly influenced, and moulded, by their prior experiences as students, as well as their observations of their own teachers. Not only are teachers’ beliefs, experiences and biographies important influences on the quality of the programs that they teach, but their beliefs and experiences as learners also play an important role in shaping their identity as teachers and for teaching [37]. This highlights the importance of transformative approaches [28] to pre-service teacher education to disrupt pre-conceived notions of school-based physical activity. Pre-service teachers who are comprehensively and positively immersed in active pedagogy, framed by transformative pedagogy [28], are potentially more likely to develop a better commitment to active teaching, possibly resulting in improved teaching and learning outcomes [36].

In regard to the changes in first year pre-service teachers’ perceptions around *Transform-Ed!*, there was a significant increase in the total scores related to their willingness to integrate active teaching and positive perceptions of activity on student outcomes, as well as their active teaching confidence and competence. There was also a significant reduction in the perceived barriers to the implementation of active teaching strategies. This is congruent with other research that has looked at specific aspects of school-based physical activity. For example, Webster and colleagues [38] reported positive changes in pre-service classroom teachers’ perceived competence and attitude towards the promotion of physical activity in school as a result of a 16-week school-based physical activity promotion course. Similarly, Webster, Erwin and Parks [39] reported positive changes in willingness to integrate movement in the academic classroom and their perceived barriers to movement integration after completion of a 16-week school physical activity promotion course. Goa et al. [40] reported a better *awareness* of organisational- or school-level barriers (e.g. lack of time or space constraint) for movement integration in schools. Similarly, McMullen and colleagues [41] noted positive outcomes in regard to pre-service physical educators’ experiences and implications in a before-school physical activity program, following initial or pre-service teacher education in this area. Although the aforementioned studies provided some positive insights into the role of physical activity-related courses in initial or pre-service teacher education programs, they were limited to mostly narrow learning experiences, for example, addressing only one component of a whole school physical activity program (e.g. only before/after school programs). In contrast, the current study incorporated a comprehensive approach to physical activity and sedentary behaviour within teacher education. Specifically, the results demonstrated a positive shift in pre-service teachers’ willingness, competence and confidence to integrate active teaching strategies within the class (i.e. active breaks, active lesson and health curriculum) as well as beyond the classroom setting (i.e. before and after school, recess and lunch time, homework). This shows that comprehensive learning experiences utilising a transformative approach [28], targeting increased physical activity and breaking sitting time can be strategically placed within initial teacher education programs to expand the development of pre-service teachers’ knowledge, skills, competence and competence to integrate activity into their current and future teaching practice.

Following the *Transform-Ed!* program, the majority of the academic educators felt more confident in their ability to integrate active teaching into seminars and lectures. The findings indicated that the *Transform-Ed!* teacher training supported all academic educators adequately. To some degree, it enabled the academic educators to ‘reimagine’ [42] themselves as teachers of active pedagogy, even teachers who initially lacked the confidence to teach in this manner. Furthermore, pre-service teachers need to be exposed to skilled academic educators who can model the teaching ‘performance’ to a high standard [43], as teachers’ beliefs about their ability to teach effectively and form meaningful connections with their students are formed early in their teaching career [44]. Therefore, it is important that pre-service teachers are mentored by academic educators experienced in active pedagogy early in their degrees.

This pilot study also demonstrated that despite some barriers to embedding *Transform-Ed!* strategies into pre-service teacher education, senior academics and principals perceived the program to be highly feasible. In addition, the potential real-world impact of the intervention at both a tertiary and primary level, such as increased reach, potential sustainability and possible institutionalisation of the changes to pre-service teacher training, was commonly shared among participants. Interventions that are effective in the long term are better suited for widespread scalability and translation and are more likely to influence policy decisions and government spending. Early and active involvement of key stakeholders (i.e. principals) and decision makers (i.e. senior academics) provides the researchers with a better understanding of the perceived barriers and facilitators to program feasibility, implementation, adherence and fidelity in real-world contexts. This understanding informs future intervention development, and may increase intervention sustainability [45], and lead to improved engagement and involvement over time [46].

Pilot trial limitations included the non-randomisation of participants into control or intervention conditions, which limited the researchers’ capacity to determine intervention effects against no treatment or control groups while other variables are kept constant. Another limitation was the isolation of embedding *Transform-Ed!* in one unit only, rather than being comprehensively embedded across all pre-service teacher education units, and scaffolded across all years of the degree. In addition, not measuring the impact of *Transform-Ed!* on primary school children’s physical activity and sedentary behaviour means there is some remaining uncertainty about the feasibility and real-world impact of this research. Future research is required to track and evaluate the impact of embedding the *Transform-Ed!* program across all years of the Bachelor of Education (Primary) degree on pre-service teachers, academics and principals’ current and future practice, utilising a randomised controlled trial. Furthermore, research should investigate the impact of the program on primary school children, specifically the impact of embedding *Transform-Ed!* into undergraduate teacher education on the pre-service teachers’ capacity to increase primary school children’s physical activity, reduce their sitting time and improve academic-related outcomes (i.e. on-task time and executive function). The continued implementation, tracking and evaluation of *Transform-Ed!* have the potential to change current teaching practices and the next generation of primary school teachers and have sustained impacts on the education system and the health of primary school students.

## Conclusion

This pilot research provided novel insights into the effects of embedding pedagogical strategies targeting physical activity into pre-service teacher education. The *Transform-Ed!* pilot study demonstrated promising results across multiple participant levels, as it was perceived as feasible, acceptable and appropriate by pre-service teachers, academics and school principals. Thus, the findings have direct implications for the expansion of the *Transform-Ed!* intervention from pilot to future definitive trial.

## Supplementary information

**Additional file 1.** The theoretical basis of the adapted version of Transform-Us! (i.e., Transform-Ed!) and links to program objectives.

**Additional file 2.** FG/Interview Guide.

**Additional file 3 **Pre-service teachers experiences of active teaching, active breaks, active homework during their own (primary) schooling years (*n* = 218).

## Data Availability

The datasets used and/or analysed during the current study are available from the corresponding author on reasonable request.
