# Supplementary material for: The feasibility and impact of embedding pedagogical strategies targeting physical activity within undergraduate teacher education: Transform-Ed!
Source: Pilot Feasibility Stud. 2019 Nov 7;5:125. doi: 10.1186/s40814-019-0507-5 (PMC6839192; doi:10.1186/s40814-019-0507-5)
Supplement: Supplementary file 1 — Additional file 1. The theoretical basis of the adapted version of Transform-Us! (i.e., Transform-Ed!) and links to program objectives. [file 40814_2019_507_MOESM1_ESM.docx]

***Additional file 1****: The theoretical basis of the adapted version of Transform-Us! (i.e., Transform-Ed!) and links to program objectives*

| **Constructs** | **Determinants** | **Program Objectives**  **(i.e., education of pre-service teachers, providing knowledge, skills, and strategies to…)** |
| --- | --- | --- |
| Intrapersonal |  |  |
| Confidence | Self-efficacy | Improve primary students’ confidence in ability to be active or reduce sedentary time |
| Preference | Enjoyment | Increase primary students’ enjoyment and preference for physical activity |
| Expectations | Benefits/barriers | Increase students’ knowledge of benefits & strategies to overcome barriers |
| Expectancies | Evaluation of anticipated outcome | Alter students’ perception of pros and cons of being more active |
| Skills | Self-management | Facilitate students self-rewards, self-instructions in factors around physical activity and sedentary behaviour (e.g., TV viewing styles) |
| Behavioral rehearsal | Self-monitoring & contracting | Assist students with goal setting, contracting with others, rewards |
| Interpersonal |  |  |
| Observational learning | Modelling | Model active teaching in all aspects of teaching.  Encourage parents & siblings to reduce their own SB & increase PA via active homework tasks |
| Social support | Modelling/social support | Encourage parents & siblings to support child to spend less time in SB & more time in PA (active homework)  Teachers encourage/support PA during recess/lunch |
| Social structure | Rules | Newsletters, block, parent information sessions to assist parents in enforcing rules regarding limiting screen time at home, during meals, during daylight hours |
| Environmental |  |  |
| Imposed environment | Availability | Increase the amount of PA equipment available at school (lunch/recess/classroom) & home.  Assist parents or increase parent knowledge (e.g., newsletters, blogs, parent evenings, homework) around reduction of the availability of TVs/computers/electronic games at home |
| Imposed environment | Access | Increase access/opportunities for PA at school & at home. Decrease access to TV/computers/electronic games at home. |
| Imposed environment | Policy | Establish class rules around compulsory interrupted sitting during class-time; ensure there is a presence of supervising teachers during recess/lunch to encourage activity |

* Adapted from Salmon et al 2011. Based on social cognitive theory (24), behavioral choice theory (25) and ecological systems theory (26)
