# Supplementary material for: The feasibility and impact of embedding pedagogical strategies targeting physical activity within undergraduate teacher education: Transform-Ed!
Source: Pilot Feasibility Stud. 2019 Nov 7;5:125. doi: 10.1186/s40814-019-0507-5 (PMC6839192; doi:10.1186/s40814-019-0507-5)
Supplement: Supplementary file 2 — Additional file 2. FG/Interview Guide. [file 40814_2019_507_MOESM2_ESM.docx]

***Additional file 2****: FG/Interview Guide*

| **Topic** | **Elaboration** | **Guide/prompt** |
| --- | --- | --- |
| Appropriateness, acceptability and feasibility of Transform-ED! | Appropriateness, acceptability and feasibility of integrated active teaching strategies in one core unit and potentially more broadly across the Bachelor of Primary Education. | Based on your experience, do you think the planned strategy is appropriate? (Prompts: why/why not/can you explain a bit further?)  What challenges do you foresee and how might they be overcome? (Prompt: anything else?)  Do you see this as a valuable inclusion to the course? (Prompts: why/why not/can you explain a bit further?) |
| Appropriateness, acceptability and feasibility of training | Appropriateness, acceptability and feasibility of training Unit Chairs, lecturers, sessional staff to integrate and deliver an adapted course | How might we best plan for sustainability so Unit chairs, lecturers and sessional staff feel confident to deliver the adapted units and have the skills required to do so? (Prompt: anything else?) |
| Potential impact | Teaching | What impact, if any, do you think this type of active teaching would have on:  The existing teaching practices within a school?  (*Prompt if needed, i.e. the way other teachers deliver their lessons*)  The wider school environment and culture?  (*Prompt if needed, i.e. the school culture and norms regarding pedagogy and importance of active classrooms or inclusion of any policies to support physical activity/reductions in prolonged sitting time*)  Would you or your teachers be likely to adopt this approach in their school? Why/why not? |
| Potential Impact | Student outcomes | Do you think active teaching strategies would have an impact, which can be either positive or negative, on child health and cognitive outcomes?  Specifically, what impact if any do you think MI would have on: learning and academic outcomes & classroom behaviour  *If yes*, can you describe this impact  *If no*, why not? |
| Potential impact | Student engagement | Do you think active teaching strategies would have any impact on children’s level of engagement and concentration during class lessons, their concentration or time-on-task?  *If yes*, can you describe this impact  *If no*, why not? |
| Potential Impact | Preservice teacher education | Can you foresee any advantages or disadvantages to training preservice teachers in the delivery of active class lessons during their University degrees? (*Prompt if needed*,  *If yes advantages*, can you describe them  *If yes disadvantages*, can you describe them |
| Barriers/facilitators to widespread integration |  | Overall, what potential barriers do you foresee that may prevent successful integration of these strategies across multiple Units? (Prompt if necessary, at an individual [teaching/staff level] and organisational [University/school] level)  How might these barriers be overcome? |
