# Supplementary material for: The feasibility and impact of embedding pedagogical strategies targeting physical activity within undergraduate teacher education: Transform-Ed!
Source: Pilot Feasibility Stud. 2019 Nov 7;5:125. doi: 10.1186/s40814-019-0507-5 (PMC6839192; doi:10.1186/s40814-019-0507-5)
Supplement: Supplementary file 3 — Additional file 3 Pre-service teachers experiences of active teaching, active breaks, active homework during their own (primary) schooling years (n = 218). [file 40814_2019_507_MOESM3_ESM.docx]

***Additional File 3****: Pre-service teachers experiences of active teaching, active breaks, active homework during their own (primary) schooling years (n=218)*

| Variable | Strongly agree/agree | Neither | Strongly disagree/disagree |
| --- | --- | --- | --- |
| Movement was incorporated regularly across the school day (not including physical education) |  | 3% | 97% |
| Classroom teachers regularly provided active teaching |  |  | 100% |
| Classroom teachers broke prolonged sitting time with active breaks | 1% |  | 99% |
| The importance of physical activity was conveyed by the generalist teacher | 2% |  | 98% |
| Activity was promoted at recess and lunch time | 16% |  | 42% |
| Active homework opportunities were provided | 1% | 1% | 98% |
